# Slow-Pathway Visualization by Using Panoramic View: A Novel Ablation Technique for Ablation of Atrioventricular Nodal Reentrant Tachycardia

**DOI:** 10.3390/jcdd9040091

**Published:** 2022-03-22

**Authors:** Lei Ding, Sixian Weng, Hongda Zhang, Fengyuan Yu, Yingjie Qi, Shu Zhang, Min Tang

**Affiliations:** Department of Cardiology, Fuwai Hospital, National Center for Cardiovascular Diseases, Chinese Academy of Medical Sciences & Peking Union Medical College, Beijing 100037, China; dingleifw@163.com (L.D.); wengzizhi@163.com (S.W.); zhanghongda213@163.com (H.Z.); fybd88@foxmail.com (F.Y.); qiyingjie0@163.com (Y.Q.); zsfuwai@vip.163.com (S.Z.)

**Keywords:** atrioventricular nodal reentrant tachycardia (AVNRT), dielectric imaging system, panoramic view, radiofrequency catheter ablation (RFCA), slow pathway (SP)

## Abstract

(1) Background: The panoramic view of a novel wide-band dielectric mapping system could show the individual anatomy. We aimed to compare the feasibility, efficacy and safety of the panoramic view guided approach for ablation of AVNRT with the conventional approach. (2) Methods: Ablation distributions in eight patients were retrospectively analyzed using the panoramic view. The para-slow-pathway (para-SP) region was divided into three regions, and the region that most frequently appeared with the appropriate junctional rhythm or eliminated the slow-pathway was defined as the adaptive slow-pathway (aSP) region. Twenty patients with AVNRT were then ablated in the aSP region under the panoramic view and compared with 40 patients using the conventional approach. (3) Results: Thirty ablation points were analyzed. The majority of effective points (95.0%) were located in the inferior and anterior portions of the para-SP region and defined as the aSP region. Baseline characteristics, fluoroscopic duration, and mean number of ablations were similar among the two groups. The panoramic view group had a significantly higher percentage of appropriate junctional rhythm (81.9% ± 26.0% vs. 55.7% ± 30.5%, *p* = 0.002) than the conventional group. (4) Conclusions: The use of the panoramic view for AVNRT ablation achieved similar clinical endpoints with higher ablation efficiency than the conventional approach.

## 1. Introduction

Atrioventricular nodal reentrant tachycardia (AVNRT) is the most common paroxysmal supraventricular tachycardia, with an incidence of 35/100,000 person-years [1]. Catheter ablation of the slow pathway (SP) has become the most effective treatment for AVNRT. Traditionally, fluoroscopy and distinctive electrograms (including Haissaguerre potential and Jackman potential [2,3]) are used to locate the SP within the triangle of Koch. However, there is still a 1.3–4% recurrence rate and approximately 1% risk of AV block reported in previous studies [4], probably due to the anatomic variations of the triangle of Koch [5,6].

Recently, a novel wide-band dielectric system (KODEX-EPD) has been introduced that provides detailed endocardial anatomy by a 3D flattened panoramic view (PANO View) [7]. Its accuracy has been verified in vitro and especially in the left atrium in human study [8]. To further assess its value in clinical practice, we hypothesized that the use of PANO View would clearly show the individual anatomic details of the para-SP region, which can improve the efficiency of slow-pathway ablation and reduce the complications of radiofrequency catheter ablation (RFCA).

## 2. Methods

The present study included two parts. The first part retrospectively analyzed the distribution of ablation targets, the frequency of junctional rhythm, and whether the SP was eliminated during ablation in eight patients with slow-fast AVNRT by PANO View (KODEX-EPD Solutions, Philips, Best, The Netherlands). The second part consisted of 20 consecutive AVNRT patients who underwent RFCA with the guidance of PANO View between September 2020 and January 2021. For comparison, in the control group, patients with AVNRT who underwent RFCA by a conventional mapping system (Ensite NavX, St. Jude Medical, St. Paul, MN, USA) were matched in a ratio of 2:1 based on sex and age. All patients signed informed consent forms before the procedure. This study was approved by the Ethics Committee of Fuwai Hospital, Chinese Academy of Medical Sciences, and was in accordance with the Declaration of Helsinki. Routine blood biochemistry, chest X-ray imaging, and cardiac echocardiography were completed in all patients before the electrophysiological study. Antiarrhythmic drugs were discontinued for at least five half-lives prior to the procedure.

A decapolar catheter (7-F steerable catheter; Triguy; APT Medical, Shenzhen, China) was cannulated in the coronary sinus (CS) via an inferior or superior approach. Two quadripolar catheters (6-F fixed catheter; Triguy; APT Medical, Shenzhen, China) were placed in the His bundle area and the right ventricle through the right femoral vein. The surface electrocardiogram (ECG) and bipolar intracardiac electrograms were continuously monitored by a digital recording system (Bard Electrophysiology Division C. R. Bard, Inc., Lowell, MA, USA). Every patient underwent programmed stimulation and diagnostic pacing maneuvers to induce tachycardia and confirm the diagnosis. In patients in whom tachycardia could not be induced by a regular procedure, isoproterenol was administered. The SP was mapped and ablated with a 4-mm nonirrigated catheter (Triguy; APT Medical, Shenzhen, China) during sinus rhythm. The endpoints of the procedure were as follows: (1) eliminated the SP (absence of AH jump or echo beat) or only a single echo beat; (2) noninducibility of AVNRT via programmed stimulation at baseline and during isoproterenol infusion. Procedure time was defined as the time from the beginning of three-dimensional (3D) reconstruction to the end of the postprocedure stimulation. Fluoroscopy time was recorded from the end of the vein puncture to the end of the procedure. The number of ablation deliveries was also recorded.

**Figure 1:**
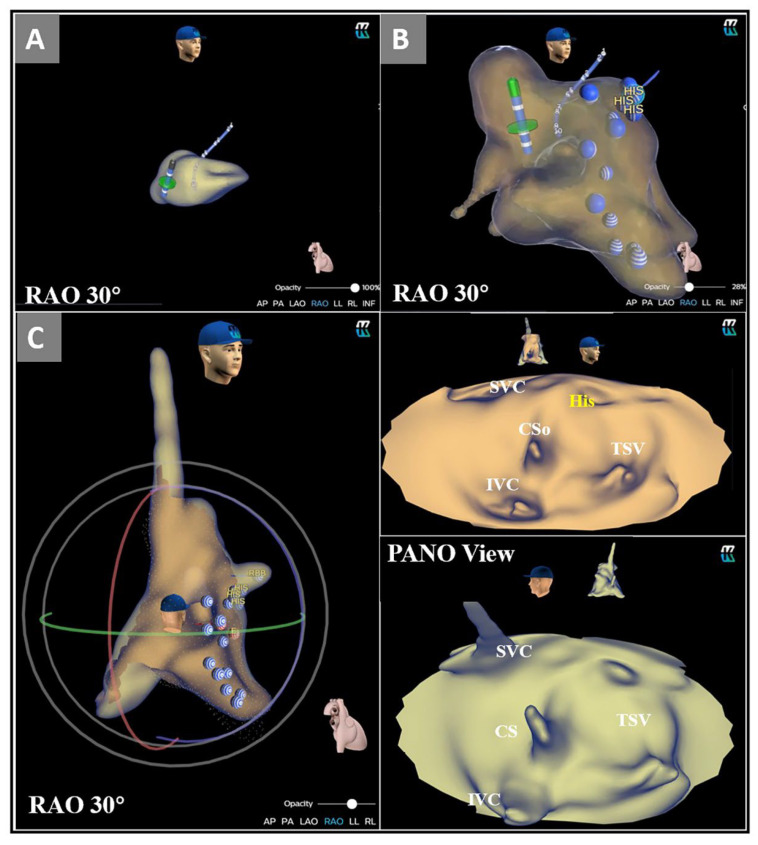
Stepwise approach to determine the para-SP region in PANO View. (**A**) The ablation catheter was advanced into the heart, and an instant image of the right atrium was created; (**B**) More details of anatomical landmarks in the right atrium were acquired; (**C**): The RAO in was chosen in the 3D reconstruction model, the heart was opened in PANO View, and the cutting line was adjusted to ensure a clear view of the para-SP region. ABL = ablation catheter; CS = coronary sinus, CSo = coronary sinus ostium; His = His bundle electrogram; IVC = inferior vena cava; LAO = left anterior oblique; PANO View = Panoramic View; RAO = right anterior oblique; SP = slow pathway; TA = tricuspid annulus.

The KODEX-EPD mapping system is a novel 3D navigation and mapping system that uses a novel di-electric imaging technology [8] and offers, in addition to a 3D image of the cardiac chambers, an innovative option, the 3D flattened PANO View. This is an unfolded projection of the chamber for the operators to visualize the anatomy of intracardiac structures. The stepwise acquisition process was as follows (Figure 1A,B). First, the ablation catheter was advanced into the heart, and an instant image of the right atrium was roughly created. At this stage, anatomical landmarks were characterized by a steep intratorso electrical field gradient as induced by the system’s set of body surface sensors. The inferior vena cava-right atrium and right atrium-superior vena cava junctions, as well as the CS ostium and the tricuspid annulus, were depicted on both the conservative 3D reconstruction (as ‘buds’) and the PANO View (as ‘dimples’). Second, a higher resolution image was created as the ablation catheter was navigated through and contacted the main structures, including the CS and the tricuspid annulus. Then, the operator would optimally preset the PANO View to facilitate self-explanatory and informative assessment of the right atrial endocardial surface by opening the right anterior oblique (RAO) projection of the 3D reconstruction model (Supplementary Video S1). Careful adjustment of the cutting plane ascertained a clear view of the para-SP region.

**Figure 2:**
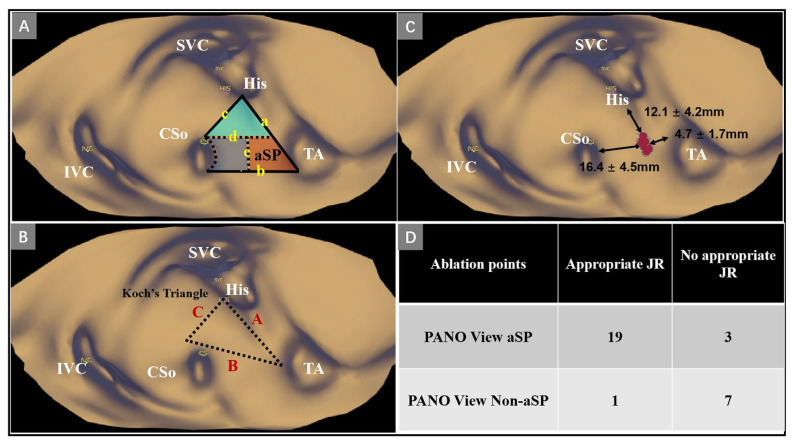
Ablation point distributions of the retrospective part. (**A**) Schematic view of the para-SP region. The para-SP region was divided into three distinct regions, which served as location sites for target distribution; a = closed line segment bound by the proximal His electrogramand tangential to the attachment line of the septal tricuspid leaflet; b = closed line segment bounded by the lowest point of the CS ostium and tangential to the CS; c = closed line segment bounded by the proximal His electrogramand the highest point of the CS ostium; d = closed line segment bounded by the highest point of the CS ostium and tangential to the CS; e = closed line segment bounded by the middle of the b and vertical to b; (**B**): Schematic view of the triangle of Koch. A = the anterior edge; B = the basal edge; C = the posterior edge; (**C**) Schematic view of effective ablation point distribution. Red dots represent ablation points; (**D**) Descriptive analysis of ablation point distribution. aSP = adaptive slow-pathway; CSo = coronary sinus ostium; His = His bundleelectrogram; IVC = inferior vena cava; JR = junctional rhythm; PANO View = Panoramic View; SVC = superior vena cava; TA = tricuspid annulus.

Eight patients with AVNRT who underwent a conventional RFCA procedure were retrospectively selected to investigate the relationship between the target distribution and ablation response. The results of the PANO View analysis were blinded to the operators and were acquired offline after the procedure. The cutting plane was carefully adjusted to expose the septal side of the right atrium (Figure 1C). The anatomic landmarks, such as the inferior vena cava, the CS ostium, and the tricuspid septal valve were first identified in the PANO View. The Koch’s triangle was precisely defined as described in previous studies [9,10] (Figure 2B). The para-SP region was divided into three distinct regions that served as location sites for ablation target distribution; more details are shown in Figure 2A. Appropriate junctional rhythm was defined as less than 120 bpm and was associated with a 1:1 VA relation according to previous studies [11,12]. The response of every ablation application, including junctional rhythm appearance, was evaluated by two experienced electrophysiologists. Finally, the relationship between the distribution of ablation targets and ablation response was summarized. The region that most frequently appeared with either appropriate junctional rhythm or elimination of the SP was defined as the adaptive slow pathway (aSP) region.

**Figure 3:**
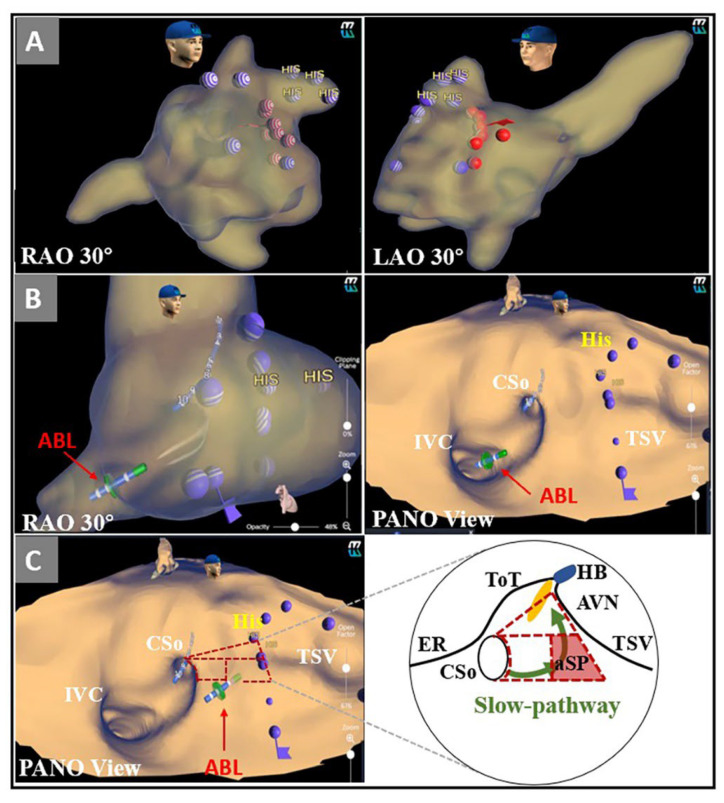
Stepwise approach to ablation of the aSP region under the guidance of PANO View. (**A**) Target distributions in RAO and LAO views; (**B**) The ablation catheter was advanced to the para-SP region under the guidance of PANO View; (**C**) The ablation catheter was targeted to the aSP region and began ablation. ABL = ablation catheter; AVN = atrioventricular node; CSo = coronary sinus ostium; ER = Eustachian ridge; HB = His bundle; IVC = inferior vena cava; LAO = left anterior oblique; PANO View = Panoramic View; RAO = right anterior oblique; ToT = tendon of Todaro; TSV = tricuspid septal valve.

Twenty patients with AVNRT were prospectively enrolled in the PANO View group. After the electrophysiology study confirmed the diagnosis of AVNRT, a nonirrigated ablation catheter with a 4 mm tip (Triguy; APT Medical, Shenzhen, China) was introduced into the right atrium, and system optimization was performed at the same time. The ablation catheter was advanced via an 8.5-F long sheath (SL1 or SR0). The stepwise approach was as follows (Figure 3B,C, Supplementary Video S2): (1) Initially, the general and detailed right atrial geometry was reconstructed by the KODEX-EPD system. The positions of the CS ostium, His bundle, and tricuspid annulus were labeled; (2) then, the PANO View was optimally arranged as previously described (Figure 1C); (3) finally, RFCA with a maximum power of 45 W and a maximum temperature of 60 °C for up to 60–90 s was delivered in the aSP region, which was determined by the retrospective part. If the appropriate junctional rhythm appeared in RFCA delivery, programmed stimulation was performed to reassess the quality of antegrade atrioventricular conduction and confirmed the noninducibility of AVNRT. If the AVNRT remained inducible or did not show the appropriate junctional rhythm in the aSP region, the ablation catheter was moved to the other regions, and RFCA deliveries were repeated with a similar stepwise approach until the appearance of the appropriate junctional rhythm or the elimination of the SP. After eliminating the SP, another reinforcing RFCA was delivered. After the whole procedure, we also measured the three edges of the triangle of Koch, the minimal distance between the inferior vena cava and CS ostium and the maximal and minimal diameters of the CS ostium. The distances between the ablation targets and the CS ostium, the tricuspid septal leaflet and the proximal His electrogram were also measured (Figure 2C).

In the control group, 3D reconstruction and mapping of the right atrium were performed with the Ensite NavX system. The sheaths, ablation catheters, electrophysiologic study procedures, RFCA parameters, and ablation endpoints were the same as those in the PANO View group. In contrast, in the control group, the RFCA target was the region where the Haissaguerre potential or the Jackman potential [2,3] was recorded. If we could not record those potentials, the bottom of the CS where atrial potential was barely recorded (A: V ≈ 1:10~1:2) in the distal bipole was also considered the target [13]. If the junctional rhythm did not appear within the initial 15 s, RFCA was stopped, and the ablation catheter was moved 2 mm higher and more anteriorly or posteriorly until the appearance of the junctional rhythm. 

Patients who underwent ablation were followed up by outpatient visits or telephone calls, and the final census date was 30 January 2022. All arrhythmia-related symptoms, including palpitations, chest tightness, chest pain, dyspnea, and syncope were recorded. Meanwhile, a 12-lead ECG was performed when patients reported any of the symptoms mentioned above. Recurrence was defined as the presence of symptoms and electrocardiographic documentation of AVNRT.

Statistical analyses were performed using SPSS IBM 22 (IBM Co., Armonk, NY, USA) and GraphPad Prism 8.0 (GraphPad Software Inc., La Jolla, CA, USA). Continuous variables were presented as the means ± standard deviation (SD) or median and interquartile range (25th–75th percentile), depending on the normality of the distribution, and were compared with a Student’s *t* test for independent samples. Categorical variables were expressed as frequency counts and percentages and compared with the chi-square test. Statistical significance was defined as *p* < 0.05.

## 3. Results

A total of thirty ablation points among eight patients were analyzed. The para-SP region was divided into three regions (Figure 2A), and the triangle of Koch was visualized in PANO View (Figure 2B). We found that the majority of effective points (19 of 20, 95.0%) were located in the inferior and anterior portions of the para-SP region (Figure 2A). In this region, nearly 90% of ablation points resulted in an appropriate junctional rhythm during ablation, while in other regions, a junctional rhythm was achieved in only one of eight ablation points (12.5%) (Figure 2D). We also measured the locations of ablation targets. The distances between ablation targets and the CS ostium, the tricuspid septal leaflet and the proximal His electrograml were 16.4 ± 4.5 mm, and 4.7 ± 1.7 mm and 12.1 ± 4.2 mm, respectively (Figure 2C).

In the second part, 60 patients with AVNRT were assigned to the PANO View group and control group at a ratio of 1:2. Distributions of age, sex, body mass index (BMI), hypertension, diabetes mellitus, and structural heart disease history showed no significance between the two groups. Although patients in the PANO View group had a higher left ventricular ejection fraction, both groups presented with normal cardiac function. In addition, the time from initial symptom onset to the first electrophysiology evaluations was much longer in the PANO View group. Patients in neither the PANO View group nor the control group had previous AVNRT ablation. The detailed baseline characteristics of both groups are shown in Table 1.

Patients in both groups were diagnosed with slow-fast AVNRT through an electrophysiology study. As shown in Supplementary Figure S1, eighteen patients (18/20, 90%) in the PANO view group had appropriate JR as a result of aSP region ablation. One patient was ablated in the region closer to the His bundle to have the “appropriate junctional rhythm”, and another patient was ablated in the region closer to the CS ostium to have the “appropriate junctional rhythm”. In the PANO View group, we found that the Jackman potential [2] or the Haissaguerrre potential [3] were observed in seventeen patients (17/20, 85%), and the mean A/V ratio of the target sites was 0.3 ± 0.3. We also measured the distance between the ablation sites and the His bundle (the site with the largest His electrogram) as well as the CS ostium using the PANO View and the Ensite NavX system, respectively (12.4 ± 3.5 mm vs. 12.3 ± 4.4 mm, *p* = 0.785; 17.2 ± 3.1 mm vs. 15.6 ± 3.2 mm, *p* = 0.001, Supplementary Table S1). As summarized in Table 2, the procedural duration, fluoroscopic duration, ablation time, and mean number of RFCA deliveries were similar in the two groups. During RFCA, the PANO View group had a significantly higher percentage of appropriate junctional rhythm (81.9% ± 26.0% vs. 55.7% ± 30.5%, *p* = 0.002) and fewer ablations were applied to display the appropriate junctional rhythm (1.4 ± 0.8 vs. 2.2 ± 2.2, *p* = 0.034). The immediate ablation success rate was 100% in both groups. However, the sign of an “AH jump” during the postablation electrophysiology study was more common in the control group (15% vs. 27.5%, *p* = 0.281). Overall, no procedure-related complications were observed.

Table 3 presents the measurements of anatomic landmarks in the right atrium. The anterior, posterior, and basal edges of the triangle of Koch were 19.6 ± 4.5 mm, 20.4 ± 4.2 mm, and 19.5 ± 5.3 mm, respectively. The mean distances between the ablation sites and the proximal Hiselectrogram, CS ostium, and tricuspid septal leaflet were 12.4 ± 1.8 mm, 14.2 ± 8.0 mm and 6.8 ± 4.3 mm, respectively.

All patients were followed up for a median of 15.2 months; two patients in the control group were identified as having recurrence by 12-lead ECG records, while all patients in the PANO View group were free of AVNRT (−95% vs. 100%, *p* = 0.309). No procedural-related complications, including cardiac effusions or atrioventricular block, were identified in either group.

## 4. Discussion

The present study evaluated the ablation distributions of typical AVNRT in PANO View and the feasibility of RFCA guided by the PANO View. The major findings are as follows:

1. Ninety-five percent of effective ablation points with appropriate junctional rhythm gathered in the aSP region in PANO View.
2. The ablation in the aSP region under PANO View more frequently resulted in the appropriate junctional rhythm and had a higher efficiency to eliminate the SP compared to the control group.
3. A stepwise procedure of ablation of the aSP region guided by PANO View showed high safety and efficacy.

In slow-fast AVNRT, the right inferior extension forms the antegrade limb of reentry in over 90% of cases. Inoue et al. [14] found that the right inferior extension is part of the atrioventricular node and close to the region between the CS ostium and the tricuspid septal leaflet. To improve the efficacy and safety of the AVNRT ablation, many operators have proposed different strategies to locate the SP. Jackman et al. [2] and Haissaguerre et al. [3] independently found that distinctive potential was always recorded at successful ablation sites and was usually located along the posteroseptal right atrium and close to the tricuspid annulus. These potentials were characterized by fractionated, multicomponent electrograms and recorded from the end of the atrial electrogram. Recently, Hale et al. [15] created a technique for the identification of SP. They found that identification of the late-activation, low-amplitude voltage in the inferior part of the triangle of Koch during sinus rhythm using a mapping system was a novel method to identify the SP. Ablation performed in this region achieved a high success rate and low complication rate. However, all of the above methods were indirect and required extensive clinical experience. Our study first introduced a simple anatomic approach that could visualize the endocardial surface and improve the spatial resolution. Under the guidance of the PANO View, operators could localize the anatomical landmarks more directly and precisely. According to the distribution of effective ablation points in the retrospective part, we summarized an aSP region that was lower than the upper line of the CS ostium and closer to the septal tricuspid leaflet (Figure 2A). Ablations under the guidance of PANO View in the aSP region showed similar clinical endpoints with a higher appropriate junctional rhythm percentage when compared with the conventional approach. This result is consistent with the autopsy study which found that the right inferior extension is close to the tricuspid annulus [14]. As for the reason, first, the percentage of accelerated junctional rhythm of the conventional group in our study was similar to previous studies [16,17]. Second, as reported by Iakobishvili, Z. [17], the duration and the cycle length of accelerated junctional rhythm was fluctuated in a large range. In our study, we only defined the “appropriate junctional rhythm” as less than 120 bpm and associated with a 1:1 VA relation, which may be stricter than in previous studies for defining the appropriate junctional rhythm. Third, operators could precisely localize the slow pathway through the PANO View, which may improve the occurrence of “appropriate junctional rhythm”. Finally, as the PANO View could directly display the aSP region, it may increase the catheter stability and guide good contact. In addition, we measured the dimensions of the triangle of Koch in PANO View and compared it with previous studies (Table 4). INOUE et al. [18] examined 50 human hearts, and their measurements of the triangle of Koch were larger than our measurements. Piotrowska et al. [6] and Panodozi et al. [19] also measured the triangle of Koch using autopsy and the Rhythmia system, respectively. Their measurements were similar to our measurements. Thus, we speculate that there is extensive anatomical variability in Koch’s triangle. Since such variations could influence the efficacy and safety of RFCA, we believe that the use of the PANO view can achieve individualized ablation and improve the efficacy and safety of the procedure.

**Figure 4:**
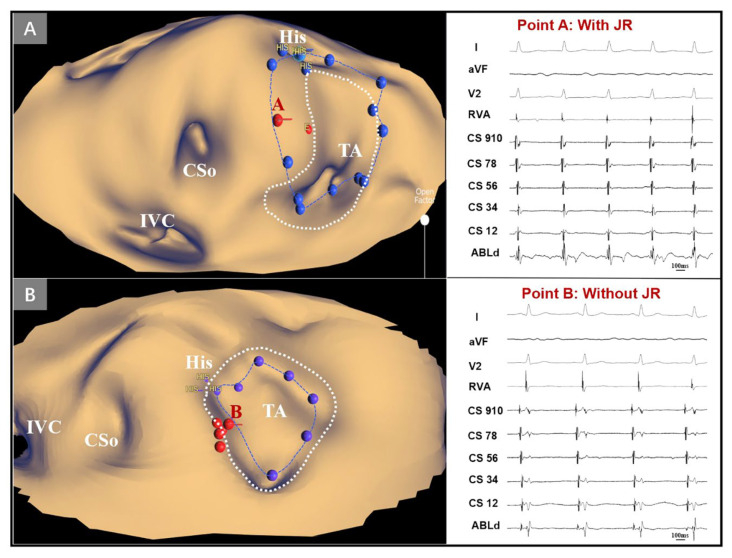
Retrospective analysis of two patients with AVNRT in PANO View. (**A**,**B**) Target distributions in PANO View. The dotted line in blue and purple represents the tricuspid annulus determined by electrograms, and the dotted line in white represents the tricuspid annulus determined by KODEX-EPD 3D reconstruction. Points A and B both represent the ablation points. Point A was located in the aSP region and exhibited the appropriate JR during ablation, while point B was located in the nonaSP region and did not exhibit JR during the whole RFCA delivery. CSo = coronary sinus ostium; His = His bundle potential; IVC = inferior vena cava; JR = junctional rhythm; PANO View = Panoramic View.

Traditionally, the determination of the tricuspid annulus is based on the “small A, large V” electrograms, but the magnitude of electrograms can be influenced by various factors, including the contacting degree, electrode space and experience of the operators [20]. Consequently, the tricuspid annulus determined only by electrograms would be different from the real location. Our PANO View approach depended on dielectric, uncontacted reconstructing technology and directly presented the intricate details of anatomic landmarks [8] with an unfolded projection. This technology delivers a real-time computed tomography (CT)-like image and determines the tricuspid annulus more accurately so that the operators can conduct a real-time anatomic ablation of the SP. For example, Figure 4 shows two tricuspid annuli in different colors that were located by electrograms and the PANO View. After the retrospective analysis, we concluded that the tricuspid annulus determined directly by the PANO View was more precise than the electrogram approach. This result also validated the accuracy and efficiency of the PANO View.

An appropriate junctional rhythm usually means that the ablation is affecting the SP and indicates successful ablation [21]. Jentzer et al. [22] analyzed 52 consecutive patients with AVNRT who underwent SP ablation and concluded that the junctional rhythm was more frequent and longer at effective sites than at ineffective sites. Iakobishvili et al. [17] also focused on the patients with AVNRT who underwent RFCA of SP and developed accelerated junctional rhythm during ablation. They compared the junctional rhythm quantity and duration between patients with or without echo beats in a postablation electrophysiology study. They finally proposed that increased quantity and duration of accelerated junctional rhythm during RFCA may be a marker of successful ablation. Our results showed that the patients who underwent ablation under the guidance of PANO View tended to have a significantly higher junctional rhythm percentage and fewer ablations required to achieve this. In addition, these results validated that the novel approach using PANO View guidance can be more efficient than the traditional approach. However, the immediate and long-term follow-up success rates did not show a significant difference in our study. We supposed that this nonsignificant result may be explained by the small sample size, and significant results may be obtained if this approach was repeated in a larger prospective population.

Regarding safety, DG et al. [13] reported that the incidence of vascular complications, AV block, and pericardial effusion was approximately 0.3%. Chrispin et al. [23] conducted a large, contemporary cohort study and concluded that complications, including AV block, were approximately 0.4%. There were no complications during the procedure or after follow-up in either group in our study. This finding is consistent with previous studies. It is clear that the guidance of PANO View in ablation of the slow pathway is a safe approach.

During the ablation of AVNRT, atrioventricular node block is still an important complication, especially for young operators. This study presents an early experience of ablation under the guidance of the PANO View. The PANO View provides a clear visualization of the para-SP region, including the triangle of Koch, and advances the orientation for educational purposes. We believe that the ablation of AVNRT will be more convenient, especially for young operators, and the incidence of atrioventricular node injuries will also be reduced. In addition, many researchers have already used the PANO View to determine the anatomic landmarks of the left atrium [7,8]; thus, this approach can be used for the guidance of transseptal puncture, catheter advancement and ablation of other complex arrhythmias in the future.

## 5. Limitations

The present study has several limitations. First, this was a single-center study with a relatively small sample size. However, the statistical power was able to assess differences in success and complication rates between the two groups. A multicenter prospective study with a larger population should be conducted to verify the safety and efficacy of ablation guided by the PANO View. Moreover, we have not yet used this technique in atypical AVNRT; however, it is conceivable that it also could be efficiently used in the left inferior extension region. In addition, the included population only consisted of patients with AVNRT, so PANO View technology should be validated in more complex arrhythmias in the future. Finally, as advanced age may be a risk factor of AV block, a group of older patients should be included to evaluate the influence of age on the anatomical morphology.

## 6. Conclusions

The use of PANO View for AVNRT ablation allows us to achieve similar clinical endpoints with higher ablation efficiency when compared with the conventional approach. This simple and novel method is expected to advance the ablation of atypical AVNRT and complex arrhythmias.

## Figures and Tables

**Table 1:** Baseline characteristics between two groups.

	All Patients (n = 60)	PANO View Group (n = 20)	Control Group (n = 40)	*p*-Value
Age (years)	48.9 ± 15.4	52.2 ± 13.8	47.3 ± 16.1	0.252
Sex, female (n, %)	33 (55.0)	13 (65.0)	20 (50.0)	0.271
BMI (n, %)	24.1 ± 3.0	24.6 ± 2.3	23.9 ± 3.4	0.397
Hypertension (n, %)	13 (21.7)	6 (30.0)	7 (17.5)	0.268
Diabetes mellitus (n, %)	8 (13.3)	3 (15.0)	5 (12.5)	0.788
Structural heart disease (n, %)	0	0	0	-
Duration from symptoms onset to first EPS (months)	60 (12.0, 120)	66 (15.8, 120)	48 (12.0, 120)	0.777
Previous AVNRT ablation (n, %)	0	0	0	-
LVEF (%)	64.5 ± 3.8	66.1 ± 4.2	63.8 ± 3.3	0.026

AVNRT = atrioventricular nodal reentrant tachycardia; BMI = body mass index; EPS = electrophysiology study; LVEF = left ventricular ejection fraction; PANO View = Panoramic View.

**Table 2:** Procedure data and outcomes between two groups.

	PANO View Group (n = 20)	Control Group (n = 40)	*p*-Value
AVNRT inducibility, n (%)	20 (100.0)	40 (100.0)	>0.999
SF-AVNRT, n (%)	20 (100.0)	40 (100.0)	>0.999
Exist other arrythmia, n (%)	0	2 (5.0)	0.309
AT, n (%)	0	1 (2.5)	0.476
PVC, n (%)	0	1 (2.5)	0.476
Procedure time, minutes	14.0 (11.0, 21.0)	16.0 (12.0, 21.0)	0.784
Fluoroscopy time, seconds	83.0 (45.5, 153.0)	87.5 (37.0, 161.5)	0.837
Ablation time (seconds)	248.0 (182.0, 374.0)	261.5 (214.8, 384.3)	0.458
Mean numbers of RFCA deliveries	5.3 ± 3.0	5.6 ± 2.9	0.871
Percentage of appropriate JR (%)	81.9 ± 26.0	55.7 ± 30.5	0.002
Number of ablations applied to display appropriate JR	1.4 ± 0.8	2.2 ± 2.2	0.034
Immediate success, n (%)	20 (100.0)	20 (100.0)	>0.999
Single echo, n (%)	2 (10.0)	4 (10.0)	>0.999
AH Jump, n (%)	3 (15.0)	11 (27.5)	0.281
Complications, n (%)	0	0	-
Pericardial effusion, n (%)	0	0	-
II–III degree of AVB, n (%)	0	0	-
Recurrence, n (%)	0	2 (5.0)	0.309

AH = atrial-Hisian interval; AT = atrial tachycardia; AVB = atrioventricular block; SF-AVNRT = slow-fast-atrioventricular nodal reentrant tachycardia; EPS = electrophysiology study; JR = junction rhythm; PANO View = Panoramic View; PVC = premature ventricular contraction; RFCA = radiofrequency catheter ablation.

**Table 3:** Measurements of the triangle of Koch and other anatomic landmarks.

	PANO View Group
Anterior edge of the triangle of Koch, mm	19.6 ± 4.5
Posterior edge of the triangle of Koch, mm	20.4 ± 4.2
Basal edge of the triangle of Koch, mm	19.5 ± 5.3
Maximal diameter of the CSo, mm	13.1 ± 1.6
Minimal diameter of the CSo, mm	10.0 ± 1.7
Distance between the IVC and the CSo, mm	19.6 ± 4.1
Distance between the target and the His, mm	12.4 ± 1.8
Distance between the target and the CSo, mm	14.2 ± 8.0
Distance between the target and the TSV, mm	6.8 ± 4.3

CSo = coronary sinus ostium; IVC = inferior vena cava; PANO View = Panoramic View; TSV = tricuspid septal valve.

**Table 4:** Comparisons of the triangle of Koch between the present study and previous studies.

Name of Authors	n	Methods	Anterior Edge (mm)	Posterior Edge (mm)	Basal Edge (mm)	Triangle Area (mm^2^)
Present study	20	PANO view	19.6 ± 4.5	20.4 ± 4.2	19.5 ± 5.3	154.8 ± 65.0
INOUE et al. [16]	50	autopsy	28.9 ± 4.5	29.4 ± 5.3	26.8 ± 3.3	-
Piotrowska et al. [6]	120	autopsy	18.0 ± 3.8	20.3 ± 4.3	18.5 ± 3.0	151.5 ± 55.8
Panodozi et al. [17]	45	Rhythmia	18.2 ± 0.3	19.9 ± 0.5	18.1 ± 0.6	150.5 ± 6.5

## Data Availability

The datasets presented in this article are not readily available because research data is confidential. Data sharing requests are required to meet the policies of the hospital and the funder. Requests to access the datasets should be directed to doctortangmin@yeah.net.

## Supplementary Materials

The following supporting information can be downloaded at: https://www.mdpi.com/article/10.3390/jcdd9040091/s1, Figure S1: Ablation sites distributions of PANO View group; Table S1: Electroanatomic parameters of the ablation sites; Video S1: Stepwise approach to show the triangle of Koch; Video S2: An example of ablation in the aSP region.
