# Supplementary material for: Slow-Pathway Visualization by Using Panoramic View: A Novel Ablation Technique for Ablation of Atrioventricular Nodal Reentrant Tachycardia
Source: J Cardiovasc Dev Dis. 2022 Mar 22;9(4):91. doi: 10.3390/jcdd9040091 (PMC9026770; doi:10.3390/jcdd9040091)
Supplement: Supplementary file 1 [file jcdd-09-00091-s001.zip › Supplementary Figure S1.pdf]

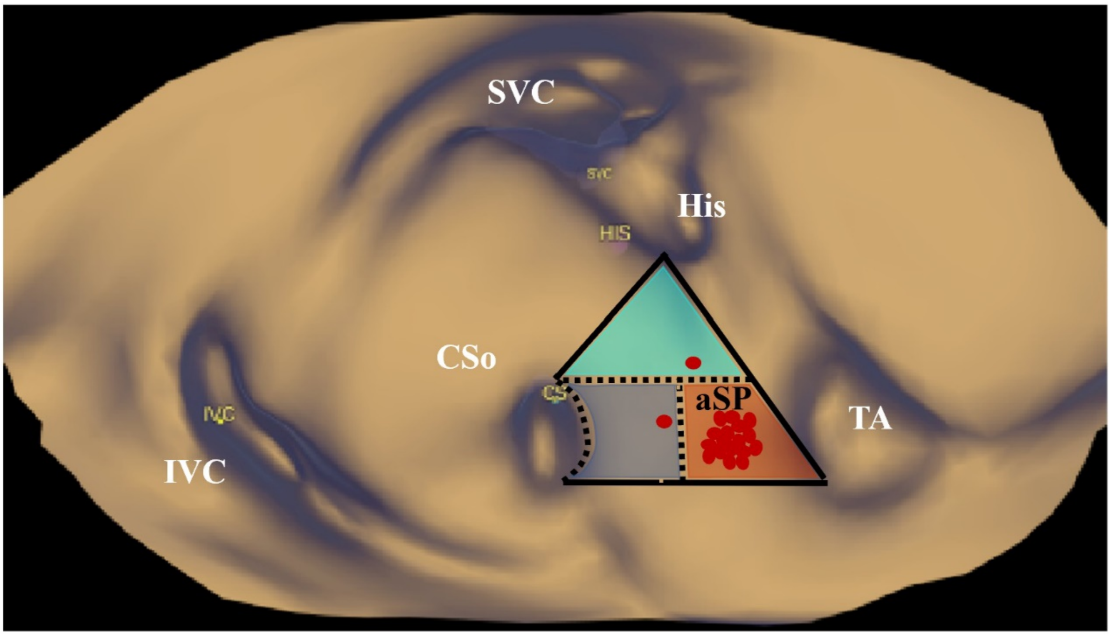

Supplementary Figure S1 Ablation sites distributions of PANO View group. Red dots represent ablation points. aSP = adaptive slow-pathway; CSo = coronary sinus ostium; His= His bundle electrogram; IVC=inferior vena cava; PANO View = Panoramic View; SVC = superior vena cava; TA = tricuspid annulus.
