# Supplementary material for: Slow-Pathway Visualization by Using Panoramic View: A Novel Ablation Technique for Ablation of Atrioventricular Nodal Reentrant Tachycardia
Source: J Cardiovasc Dev Dis. 2022 Mar 22;9(4):91. doi: 10.3390/jcdd9040091 (PMC9026770; doi:10.3390/jcdd9040091)
Supplement: Supplementary file 1 [file jcdd-09-00091-s001.zip › Supplementary Table S1.pdf]

Supplementary Table S1 Electroanatomic parameters of the ablation sites

	PANO View group	Control group	P value
Distance between ablation sites and *the His bundle (mm)	12.4 ± 3.5	12.3 ± 4.4	0.785
Distance between ablation sites and the CSo (mm)	17.2 ± 3.1	15.6± 3.2	0.001*
A/V ratio	0.3 ± 0.3	-	-

CSo = coronary sinus ostium; PANO View = Panoramic View.

*: the site with the largest His electrogram
